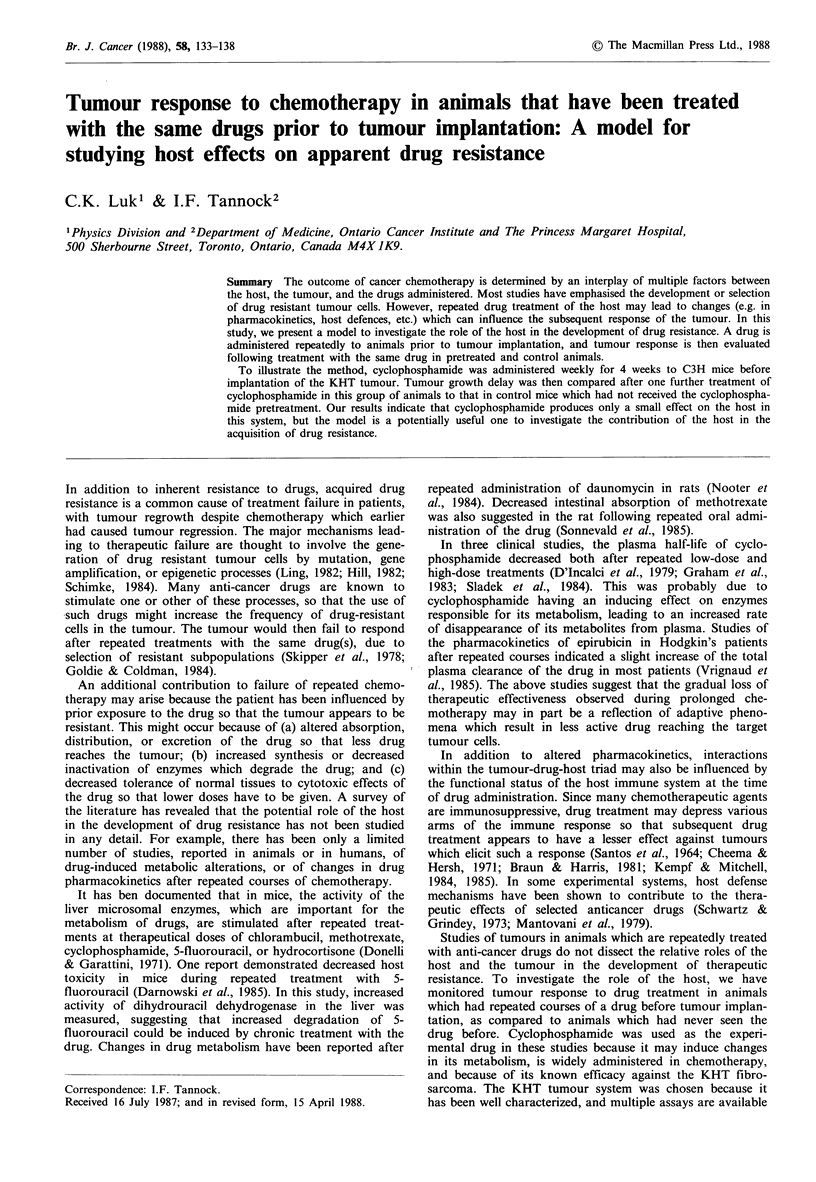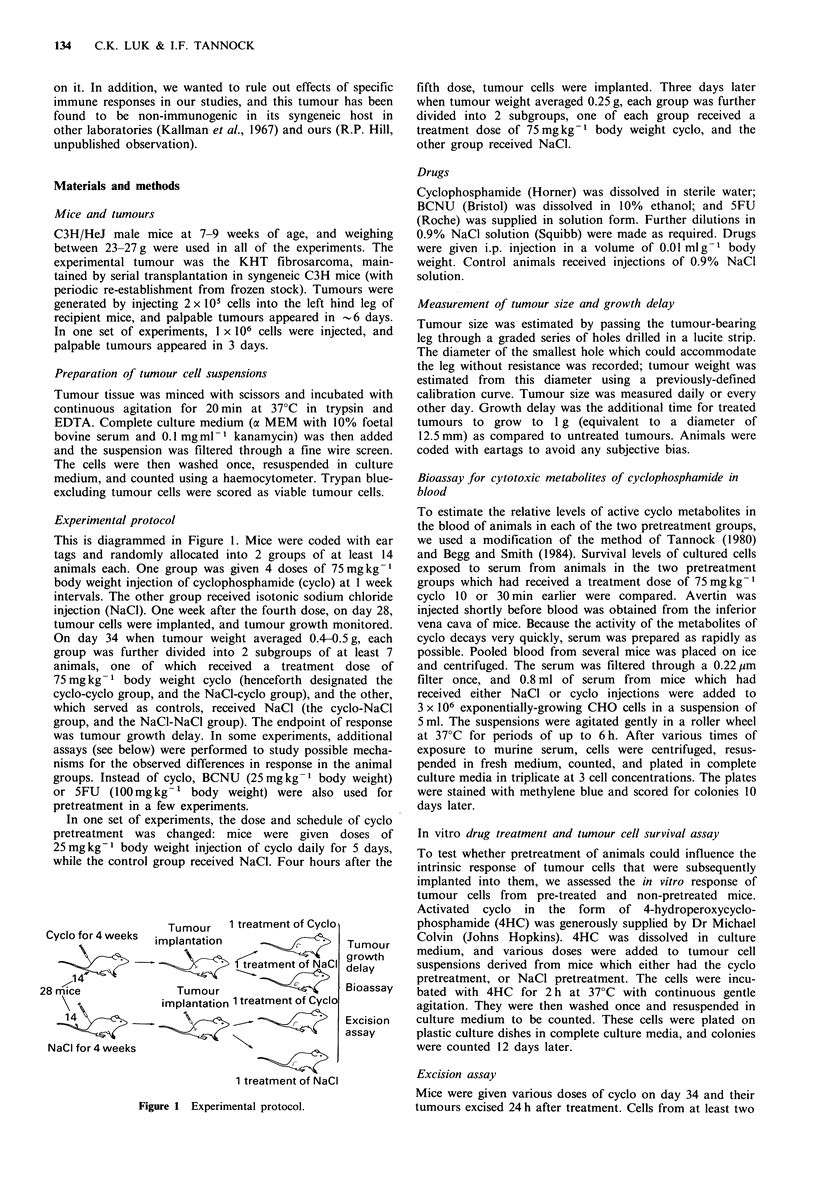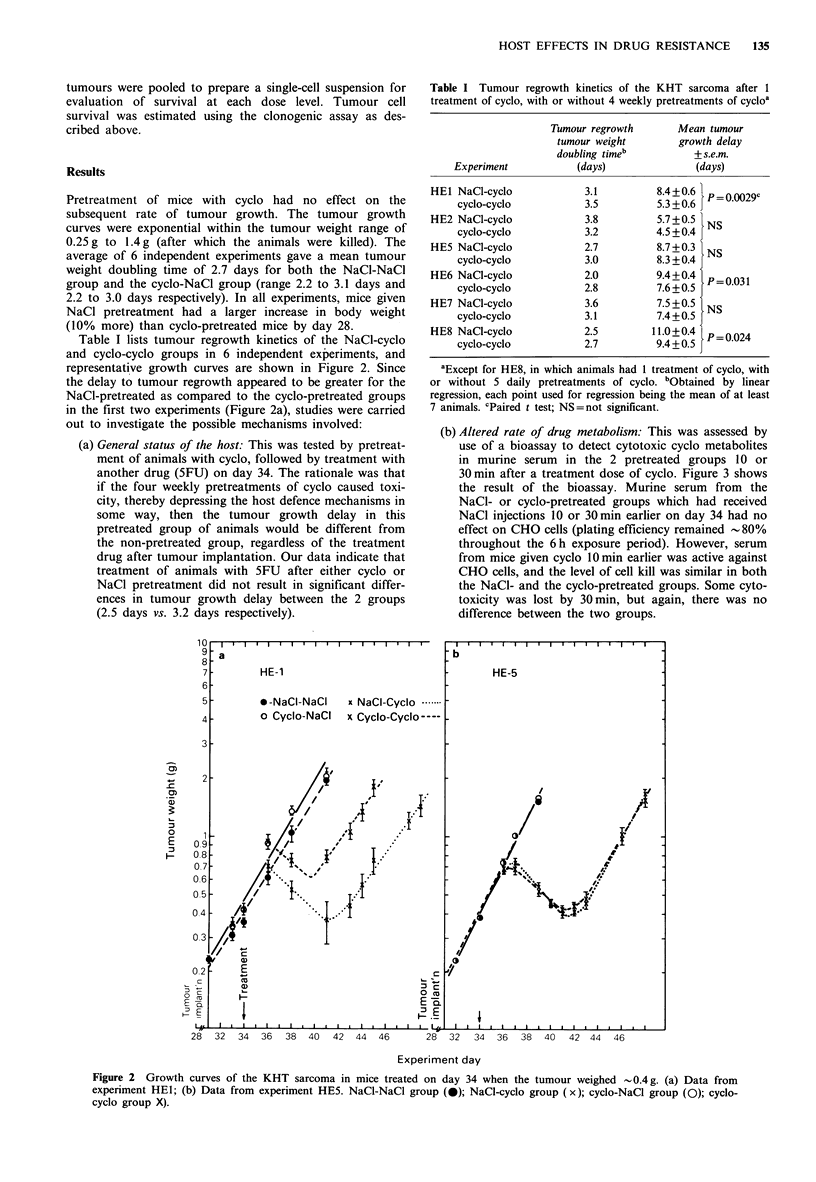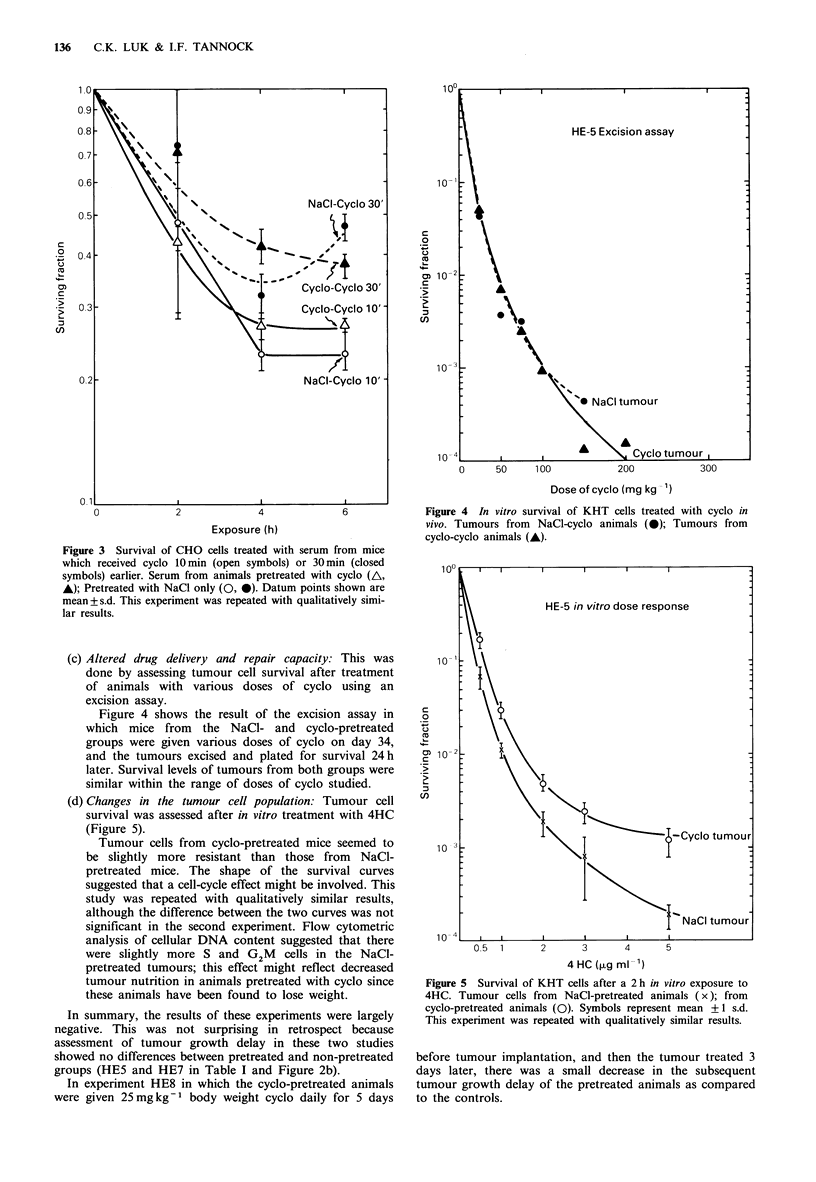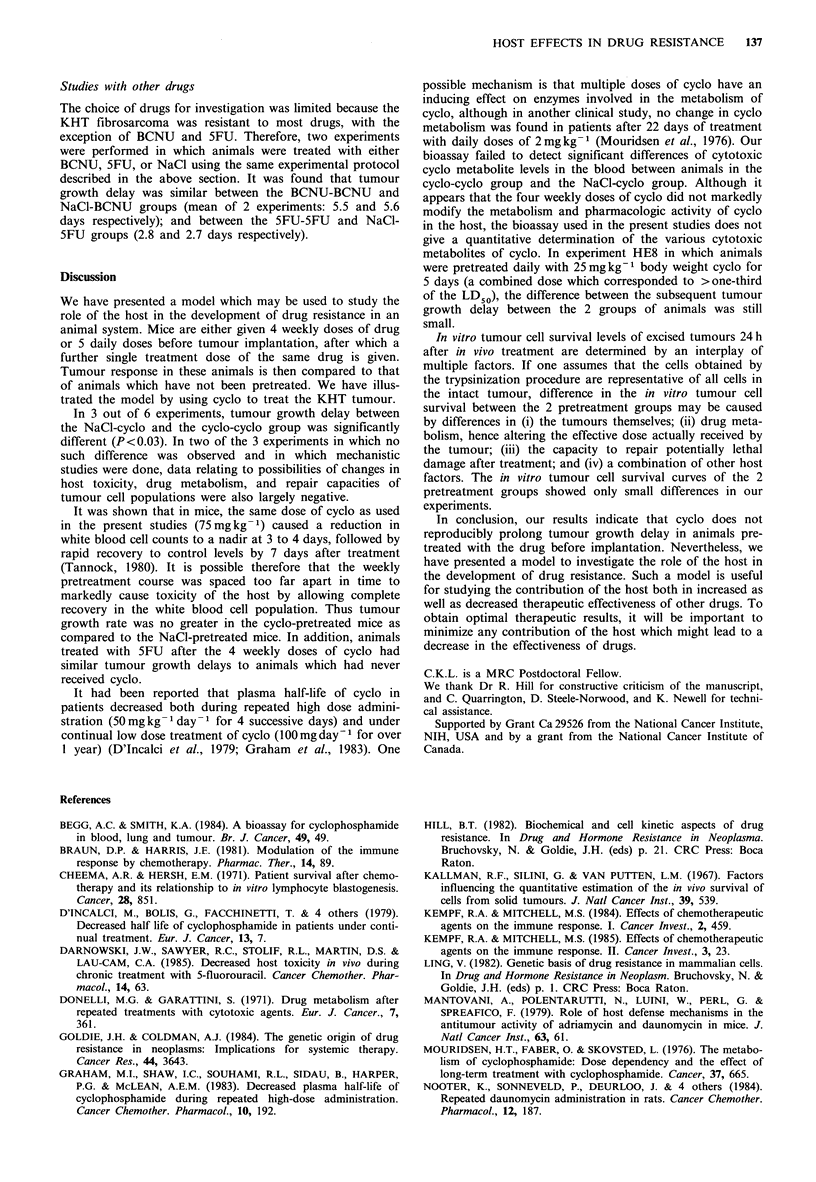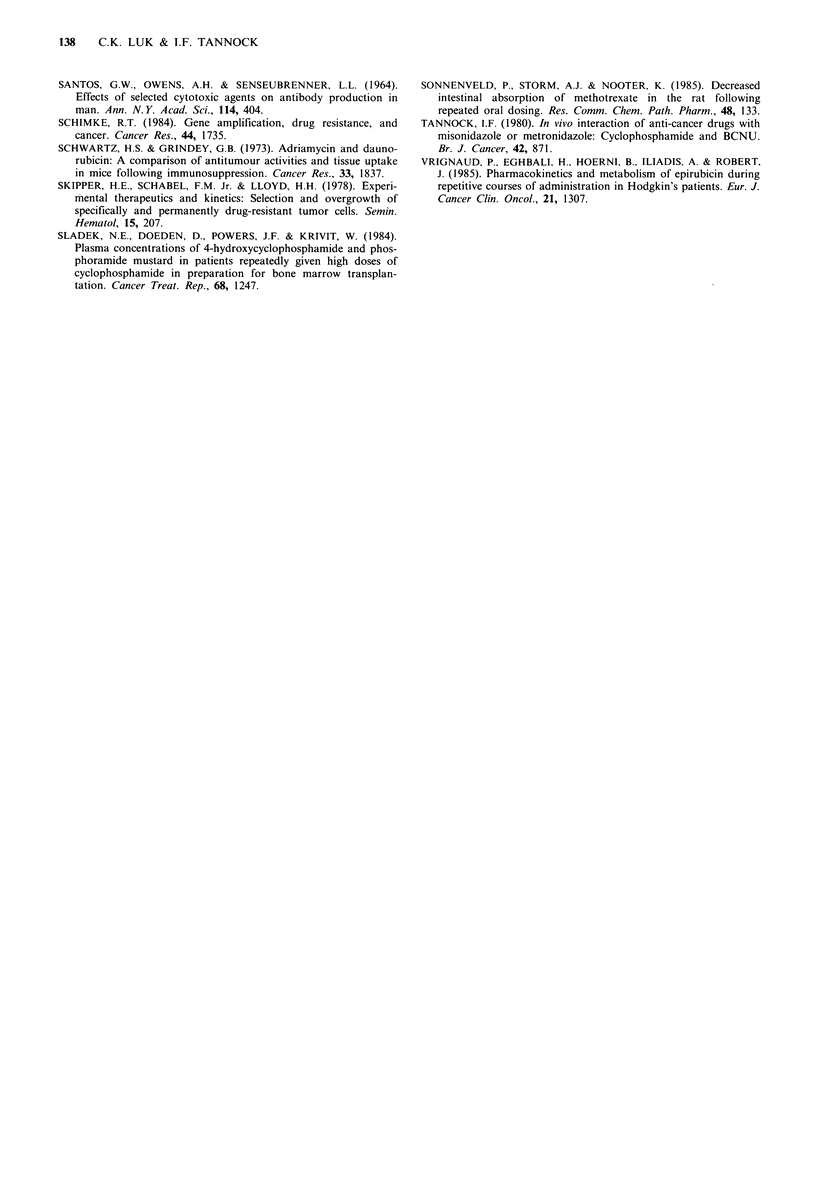# Tumour response to chemotherapy in animals that have been treated with the same drugs prior to tumour implantation: a model for studying host effects on apparent drug resistance.

**DOI:** 10.1038/bjc.1988.179

**Published:** 1988-08

**Authors:** C. K. Luk, I. F. Tannock

**Affiliations:** Physics Division, Ontario Cancer Institute, Toronto, Canada.

## Abstract

The outcome of cancer chemotherapy is determined by an interplay of multiple factors between the host, the tumour, and the drugs administered. Most studies have emphasised the development or selection of drug resistant tumour cells. However, repeated drug treatment of the host may lead to changes (e.g. in pharmacokinetics, host defences, etc.) which can influence the subsequent response of the tumour. In this study, we present a model to investigate the role of the host in the development of drug resistance. A drug is administered repeatedly to animals prior to tumour implantation, and tumour response is then evaluated following treatment with the same drug in pretreated and control animals. To illustrate the method, cyclophosphamide was administered weekly for 4 weeks to C3H mice before implantation of the KHT tumour. Tumour growth delay was then compared after one further treatment of cyclophosphamide in this group of animals to that in control mice which had not received the cyclophosphamide pretreatment. Our results indicate that cyclophosphamide produces only a small effect on the host in this system, but the model is a potentially useful one to investigate the contribution of the host in the acquisition of drug resistance.


Br. J. Cancer (1988), 58, 133–138 © The Macmillan Press Ltd., 1988

### Tumour response to chemotherapy in animals that have been treated with the same drugs prior to tumour implantation: A model for studying host effects on apparent drug resistance

C.K. Luk[1] & I.F. Tannock[2]

[1]*Physics Division and* [2]*Department of Medicine, Ontario Cancer Institute and The Princess Margaret Hospital, 500 Sherbourne Street, Toronto, Ontario, Canada M4X 1K9.*

**Summary** The outcome of cancer chemotherapy is determined by an interplay of multiple factors between the host, the tumour, and the drugs administered. Most studies have emphasised the development or selection of drug resistant tumour cells. However, repeated drug treatment of the host may lead to changes (e.g. in pharmacokinetics, host defences, etc.) which can influence the subsequent response of the tumour. In this study, we present a model to investigate the role of the host in the development of drug resistance. A drug is administered repeatedly to animals prior to tumour implantation, and tumour response is then evaluated following treatment with the same drug in pretreated and control animals.

To illustrate the method, cyclophosphamide was administered weekly for 4 weeks to C3H mice before implantation of the KHT tumour. Tumour growth delay was then compared after one further treatment of cyclophosphamide in this group of animals to that in control mice which had not received the cyclophosphamide pretreatment. Our results indicate that cyclophosphamide produces only a small effect on the host in this system, but the model is a potentially useful one to investigate the contribution of the host in the acquisition of drug resistance.

In addition to inherent resistance to drugs, acquired drug resistance is a common cause of treatment failure in patients, with tumour regrowth despite chemotherapy which earlier had caused tumour regression. The major mechanisms leading to therapeutic failure are thought to involve the generation of drug resistant tumour cells by mutation, gene amplification, or epigenetic processes (Ling, 1982; Hill, 1982; Schimke, 1984). Many anti-cancer drugs are known to stimulate one or other of these processes, so that the use of such drugs might increase the frequency of drug-resistant cells in the tumour. The tumour would then fail to respond after repeated treatments with the same drug(s), due to selection of resistant subpopulations (Skipper *et al.*, 1978; Goldie & Coldman, 1984).

An additional contribution to failure of repeated chemotherapy may arise because the patient has been influenced by prior exposure to the drug so that the tumour appears to be resistant. This might occur because of (a) altered absorption, distribution, or excretion of the drug so that less drug reaches the tumour; (b) increased synthesis or decreased inactivation of enzymes which degrade the drug; and (c) decreased tolerance of normal tissues to cytotoxic effects of the drug so that lower doses have to be given. A survey of the literature has revealed that the potential role of the host in the development of drug resistance has not been studied in any detail. For example, there has been only a limited number of studies, reported in animals or in humans, of drug-induced metabolic alterations, or of changes in drug pharmacokinetics after repeated courses of chemotherapy.

It has ben documented that in mice, the activity of the liver microsomal enzymes, which are important for the metabolism of drugs, are stimulated after repeated treatments at therapeutical doses of chlorambucil, methotrexate, cyclophosphamide, 5-fluorouracil, or hydrocortisone (Donelli & Garattini, 1971). One report demonstrated decreased host toxicity in mice during repeated treatment with 5-fluorouracil (Darnowski *et al.*, 1985). In this study, increased activity of dihydrouracil dehydrogenase in the liver was measured, suggesting that increased degradation of 5-fluorouracil could be induced by chronic treatment with the drug. Changes in drug metabolism have been reported after repeated administration of daunomycin in rats (Nooter *et al.*, 1984). Decreased intestinal absorption of methotrexate was also suggested in the rat following repeated oral administration of the drug (Sonnevald *et al.*, 1985).

In three clinical studies, the plasma half-life of cyclophosphamide decreased both after repeated low-dose and high-dose treatments (D'Incalci *et al.*, 1979; Graham *et al.*, 1983; Sladek *et al.*, 1984). This was probably due to cyclophosphamide having an inducing effect on enzymes responsible for its metabolism, leading to an increased rate of disappearance of its metabolites from plasma. Studies of the pharmacokinetics of epirubicin in Hodgkin's patients after repeated courses indicated a slight increase of the total plasma clearance of the drug in most patients (Vrignaud *et al.*, 1985). The above studies suggest that the gradual loss of therapeutic effectiveness observed during prolonged chemotherapy may in part be a reflection of adaptive phenomena which result in less active drug reaching the target tumour cells.

In addition to altered pharmacokinetics, interactions within the tumour-drug-host triad may also be influenced by the functional status of the host immune system at the time of drug administration. Since many chemotherapeutic agents are immunosuppressive, drug treatment may depress various arms of the immune response so that subsequent drug treatment appears to have a lesser effect against tumours which elicit such a response (Santos *et al.*, 1964; Cheema & Hersh, 1971; Braun & Harris, 1981; Kempf & Mitchell, 1984, 1985). In some experimental systems, host defense mechanisms have been shown to contribute to the therapeutic effects of selected anticancer drugs (Schwartz & Grindey, 1973; Mantovani *et al.*, 1979).

Studies of tumours in animals which are repeatedly treated with anti-cancer drugs do not dissect the relative roles of the host and the tumour in the development of therapeutic resistance. To investigate the role of the host, we have monitored tumour response to drug treatment in animals which had repeated courses of a drug before tumour implantation, as compared to animals which had never seen the drug before. Cyclophosphamide was used as the experimental drug in these studies because it may induce changes in its metabolism, is widely administered in chemotherapy, and because of its known efficacy against the KHT fibrosarcoma. The KHT tumour system was chosen because it has been well characterized, and multiple assays are available

Correspondence: I.F. Tannock.
Received 16 July 1987; and in revised form, 15 April 1988.

on it. In addition, we wanted to rule out effects of specific immune responses in our studies, and this tumour has been found to be non-immunogenic in its syngeneic host in other laboratories (Kallman *et al.*, 1967) and ours (R.P. Hill, unpublished observation).

### Materials and methods

### *Mice and tumours*

C3H/HeJ male mice at 7–9 weeks of age, and weighing between 23–27 g were used in all of the experiments. The experimental tumour was the KHT fibrosarcoma, maintained by serial transplantation in syngeneic C3H mice (with periodic re-establishment from frozen stock). Tumours were generated by injecting $2 \times 10^5$ cells into the left hind leg of recipient mice, and palpable tumours appeared in ~6 days. In one set of experiments, $1 \times 10^6$ cells were injected, and palpable tumours appeared in 3 days.

### *Preparation of tumour cell suspensions*

Tumour tissue was minced with scissors and incubated with continuous agitation for 20 min at 37°C in trypsin and EDTA. Complete culture medium (α MEM with 10% foetal bovine serum and $0.1 \text{ mg ml}^{-1}$ kanamycin) was then added and the suspension was filtered through a fine wire screen. The cells were then washed once, resuspended in culture medium, and counted using a haemocytometer. Trypan blue-excluding tumour cells were scored as viable tumour cells.

### *Experimental protocol*

This is diagrammed in Figure 1. Mice were coded with ear tags and randomly allocated into 2 groups of at least 14 animals each. One group was given 4 doses of $75 \text{ mg kg}^{-1}$ body weight injection of cyclophosphamide (cyclo) at 1 week intervals. The other group received isotonic sodium chloride injection (NaCl). One week after the fourth dose, on day 28, tumour cells were implanted, and tumour growth monitored. On day 34 when tumour weight averaged 0.4–0.5 g, each group was further divided into 2 subgroups of at least 7 animals, one of which received a treatment dose of $75 \text{ mg kg}^{-1}$ body weight cyclo (henceforth designated the cyclo-cyclo group, and the NaCl-cyclo group), and the other, which served as controls, received NaCl (the cyclo-NaCl group, and the NaCl-NaCl group). The endpoint of response was tumour growth delay. In some experiments, additional assays (see below) were performed to study possible mechanisms for the observed differences in response in the animal groups. Instead of cyclo, BCNU ($25 \text{ mg kg}^{-1}$ body weight) or 5FU ($100 \text{ mg kg}^{-1}$ body weight) were also used for pretreatment in a few experiments.

In one set of experiments, the dose and schedule of cyclo pretreatment was changed: mice were given doses of $25 \text{ mg kg}^{-1}$ body weight injection of cyclo daily for 5 days, while the control group received NaCl. Four hours after the fifth dose, tumour cells were implanted. Three days later when tumour weight averaged 0.25 g, each group was further divided into 2 subgroups, one of each group received a treatment dose of $75 \text{ mg kg}^{-1}$ body weight cyclo, and the other group received NaCl.

**Figure 1** Experimental protocol.

### *Drugs*

Cyclophosphamide (Horner) was dissolved in sterile water; BCNU (Bristol) was dissolved in 10% ethanol; and 5FU (Roche) was supplied in solution form. Further dilutions in 0.9% NaCl solution (Squibb) were made as required. Drugs were given i.p. injection in a volume of $0.01 \text{ ml g}^{-1}$ body weight. Control animals received injections of 0.9% NaCl solution.

### *Measurement of tumour size and growth delay*

Tumour size was estimated by passing the tumour-bearing leg through a graded series of holes drilled in a lucite strip. The diameter of the smallest hole which could accommodate the leg without resistance was recorded; tumour weight was estimated from this diameter using a previously-defined calibration curve. Tumour size was measured daily or every other day. Growth delay was the additional time for treated tumours to grow to 1 g (equivalent to a diameter of 12.5 mm) as compared to untreated tumours. Animals were coded with eartags to avoid any subjective bias.

### *Bioassay for cytotoxic metabolites of cyclophosphamide in blood*

To estimate the relative levels of active cyclo metabolites in the blood of animals in each of the two pretreatment groups, we used a modification of the method of Tannock (1980) and Begg and Smith (1984). Survival levels of cultured cells exposed to serum from animals in the two pretreatment groups which had received a treatment dose of $75 \text{ mg kg}^{-1}$ cyclo 10 or 30 min earlier were compared. Avertin was injected shortly before blood was obtained from the inferior vena cava of mice. Because the activity of the metabolites of cyclo decays very quickly, serum was prepared as rapidly as possible. Pooled blood from several mice was placed on ice and centrifuged. The serum was filtered through a 0.22 μm filter once, and 0.8 ml of serum from mice which had received either NaCl or cyclo injections were added to $3 \times 10^6$ exponentially-growing CHO cells in a suspension of 5 ml. The suspensions were agitated gently in a roller wheel at 37°C for periods of up to 6 h. After various times of exposure to murine serum, cells were centrifuged, resuspended in fresh medium, counted, and plated in complete culture media in triplicate at 3 cell concentrations. The plates were stained with methylene blue and scored for colonies 10 days later.

### In vitro *drug treatment and tumour cell survival assay*

To test whether pretreatment of animals could influence the intrinsic response of tumour cells that were subsequently implanted into them, we assessed the *in vitro* response of tumour cells from pre-treated and non-pretreated mice. Activated cyclo in the form of 4-hydroperoxycyclophosphamide (4HC) was generously supplied by Dr Michael Colvin (Johns Hopkins). 4HC was dissolved in culture medium, and various doses were added to tumour cell suspensions derived from mice which either had the cyclo pretreatment, or NaCl pretreatment. The cells were incubated with 4HC for 2 h at 37°C with continuous gentle agitation. They were then washed once and resuspended in culture medium to be counted. These cells were plated on plastic culture dishes in complete culture media, and colonies were counted 12 days later.

### *Excision assay*

Mice were given various doses of cyclo on day 34 and their tumours excised 24 h after treatment. Cells from at least two tumours were pooled to prepare a single-cell suspension for evaluation of survival at each dose level. Tumour cell survival was estimated using the clonogenic assay as described above.

### Results

Pretreatment of mice with cyclo had no effect on the subsequent rate of tumour growth. The tumour growth curves were exponential within the tumour weight range of 0.25 g to 1.4 g (after which the animals were killed). The average of 6 independent experiments gave a mean tumour weight doubling time of 2.7 days for both the NaCl-NaCl group and the cyclo-NaCl group (range 2.2 to 3.1 days and 2.2 to 3.0 days respectively). In all experiments, mice given NaCl pretreatment had a larger increase in body weight (10% more) than cyclo-pretreated mice by day 28.

Table I lists tumour regrowth kinetics of the NaCl-cyclo and cyclo-cyclo groups in 6 independent experiments, and representative growth curves are shown in Figure 2. Since the delay to tumour regrowth appeared to be greater for the NaCl-pretreated as compared to the cyclo-pretreated groups in the first two experiments (Figure 2a), studies were carried out to investigate the possible mechanisms involved:

(a) *General status of the host:* This was tested by pretreatment of animals with cyclo, followed by treatment with another drug (5FU) on day 34. The rationale was that if the four weekly pretreatments of cyclo caused toxicity, thereby depressing the host defence mechanisms in some way, then the tumour growth delay in this pretreated group of animals would be different from the non-pretreated group, regardless of the treatment drug after tumour implantation. Our data indicate that treatment of animals with 5FU after either cyclo or NaCl pretreatment did not result in significant differences in tumour growth delay between the 2 groups (2.5 days *vs*. 3.2 days respectively).

**Table I** Tumour regrowth kinetics of the KHT sarcoma after 1 treatment of cyclo, with or without 4 weekly pretreatments of cyclo[a]

| *Experiment* | *Tumour regrowth tumour weight doubling time*[b] *(days)* | *Mean tumour growth delay ±s.e.m. (days)* | |
|---|---|---|---|
| HE1 NaCl-cyclo | 3.1 | 8.4±0.6 | $P$=0.0029[c] |
| cyclo-cyclo | 3.5 | 5.3±0.6 | |
| HE2 NaCl-cyclo | 3.8 | 5.7±0.5 | NS |
| cyclo-cyclo | 3.2 | 4.5±0.4 | |
| HE5 NaCl-cyclo | 2.7 | 8.7±0.3 | NS |
| cyclo-cyclo | 3.0 | 8.3±0.4 | |
| HE6 NaCl-cyclo | 2.0 | 9.4±0.4 | $P$=0.031 |
| cyclo-cyclo | 2.8 | 7.6±0.5 | |
| HE7 NaCl-cyclo | 3.6 | 7.5±0.5 | NS |
| cyclo-cyclo | 3.1 | 7.4±0.5 | |
| HE8 NaCl-cyclo | 2.5 | 11.0±0.4 | $P$=0.024 |
| cyclo-cyclo | 2.7 | 9.4±0.5 | |

[a]Except for HE8, in which animals had 1 treatment of cyclo, with or without 5 daily pretreatments of cyclo. [b]Obtained by linear regression, each point used for regression being the mean of at least 7 animals. [c]Paired $t$ test; NS = not significant.

(b) *Altered rate of drug metabolism:* This was assessed by use of a bioassay to detect cytotoxic cyclo metabolites in murine serum in the 2 pretreated groups 10 or 30 min after a treatment dose of cyclo. Figure 3 shows the result of the bioassay. Murine serum from the NaCl- or cyclo-pretreated groups which had received NaCl injections 10 or 30 min earlier on day 34 had no effect on CHO cells (plating efficiency remained ~80% throughout the 6 h exposure period). However, serum from mice given cyclo 10 min earlier was active against CHO cells, and the level of cell kill was similar in both the NaCl- and the cyclo-pretreated groups. Some cytotoxicity was lost by 30 min, but again, there was no difference between the two groups.

**Figure 2** Growth curves of the KHT sarcoma in mice treated on day 34 when the tumour weighed ~0.4 g. (a) Data from experiment HE1; (b) Data from experiment HE5. NaCl-NaCl group (●); NaCl-cyclo group (×); cyclo-NaCl group (○); cyclo-cyclo group X).

**Figure 3** Survival of CHO cells treated with serum from mice which received cyclo 10 min (open symbols) or 30 min (closed symbols) earlier. Serum from animals pretreated with cyclo (△, ▲); Pretreated with NaCl only (○, ●). Datum points shown are mean ± s.d. This experiment was repeated with qualitatively similar results.

(c) *Altered drug delivery and repair capacity:* This was done by assessing tumour cell survival after treatment of animals with various doses of cyclo using an excision assay.

Figure 4 shows the result of the excision assay in which mice from the NaCl- and cyclo-pretreated groups were given various doses of cyclo on day 34, and the tumours excised and plated for survival 24 h later. Survival levels of tumours from both groups were similar within the range of doses of cyclo studied.

(d) *Changes in the tumour cell population:* Tumour cell survival was assessed after *in vitro* treatment with 4HC (Figure 5).

Tumour cells from cyclo-pretreated mice seemed to be slightly more resistant than those from NaCl-pretreated mice. The shape of the survival curves suggested that a cell-cycle effect might be involved. This study was repeated with qualitatively similar results, although the difference between the two curves was not significant in the second experiment. Flow cytometric analysis of cellular DNA content suggested that there were slightly more S and $G_2M$ cells in the NaCl-pretreated tumours; this effect might reflect decreased tumour nutrition in animals pretreated with cyclo since these animals have been found to lose weight.

In summary, the results of these experiments were largely negative. This was not surprising in retrospect because assessment of tumour growth delay in these two studies showed no differences between pretreated and non-pretreated groups (HE5 and HE7 in Table I and Figure 2b).

In experiment HE8 in which the cyclo-pretreated animals were given 25 mg kg$^{-1}$ body weight cyclo daily for 5 days before tumour implantation, and then the tumour treated 3 days later, there was a small decrease in the subsequent tumour growth delay of the pretreated animals as compared to the controls.

**Figure 4** *In vitro* survival of KHT cells treated with cyclo *in vivo*. Tumours from NaCl-cyclo animals (●); Tumours from cyclo-cyclo animals (▲).

**Figure 5** Survival of KHT cells after a 2 h *in vitro* exposure to 4HC. Tumour cells from NaCl-pretreated animals (×); from cyclo-pretreated animals (○). Symbols represent mean ±1 s.d. This experiment was repeated with qualitatively similar results.

*Studies with other drugs*

The choice of drugs for investigation was limited because the KHT fibrosarcoma was resistant to most drugs, with the exception of BCNU and 5FU. Therefore, two experiments were performed in which animals were treated with either BCNU, 5FU, or NaCl using the same experimental protocol described in the above section. It was found that tumour growth delay was similar between the BCNU-BCNU and NaCl-BCNU groups (mean of 2 experiments: 5.5 and 5.6 days respectively); and between the 5FU-5FU and NaCl-5FU groups (2.8 and 2.7 days respectively).

### Discussion

We have presented a model which may be used to study the role of the host in the development of drug resistance in an animal system. Mice are either given 4 weekly doses of drug or 5 daily doses before tumour implantation, after which a further single treatment dose of the same drug is given. Tumour response in these animals is then compared to that of animals which have not been pretreated. We have illustrated the model by using cyclo to treat the KHT tumour.

In 3 out of 6 experiments, tumour growth delay between the NaCl-cyclo and the cyclo-cyclo group was significantly different ($P<0.03$). In two of the 3 experiments in which no such difference was observed and in which mechanistic studies were done, data relating to possibilities of changes in host toxicity, drug metabolism, and repair capacities of tumour cell populations were also largely negative.

It was shown that in mice, the same dose of cyclo as used in the present studies ($75\,mg\,kg^{-1}$) caused a reduction in white blood cell counts to a nadir at 3 to 4 days, followed by rapid recovery to control levels by 7 days after treatment (Tannock, 1980). It is possible therefore that the weekly pretreatment course was spaced too far apart in time to markedly cause toxicity of the host by allowing complete recovery in the white blood cell population. Thus tumour growth rate was no greater in the cyclo-pretreated mice as compared to the NaCl-pretreated mice. In addition, animals treated with 5FU after the 4 weekly doses of cyclo had similar tumour growth delays to animals which had never received cyclo.

It had been reported that plasma half-life of cyclo in patients decreased both during repeated high dose administration ($50\,mg\,kg^{-1}\,day^{-1}$ for 4 successive days) and under continual low dose treatment of cyclo ($100\,mg\,day^{-1}$ for over 1 year) (D'Incalci *et al.*, 1979; Graham *et al.*, 1983). One possible mechanism is that multiple doses of cyclo have an inducing effect on enzymes involved in the metabolism of cyclo, although in another clinical study, no change in cyclo metabolism was found in patients after 22 days of treatment with daily doses of $2\,mg\,kg^{-1}$ (Mouridsen *et al.*, 1976). Our bioassay failed to detect significant differences of cytotoxic cyclo metabolite levels in the blood between animals in the cyclo-cyclo group and the NaCl-cyclo group. Although it appears that the four weekly doses of cyclo did not markedly modify the metabolism and pharmacologic activity of cyclo in the host, the bioassay used in the present studies does not give a quantitative determination of the various cytotoxic metabolites of cyclo. In experiment HE8 in which animals were pretreated daily with $25\,mg\,kg^{-1}$ body weight cyclo for 5 days (a combined dose which corresponded to >one-third of the $LD_{50}$), the difference between the subsequent tumour growth delay between the 2 groups of animals was still small.

*In vitro* tumour cell survival levels of excised tumours 24 h after *in vivo* treatment are determined by an interplay of multiple factors. If one assumes that the cells obtained by the trypsinization procedure are representative of all cells in the intact tumour, difference in the *in vitro* tumour cell survival between the 2 pretreatment groups may be caused by differences in (i) the tumours themselves; (ii) drug metabolism, hence altering the effective dose actually received by the tumour; (iii) the capacity to repair potentially lethal damage after treatment; and (iv) a combination of other host factors. The *in vitro* tumour cell survival curves of the 2 pretreatment groups showed only small differences in our experiments.

In conclusion, our results indicate that cyclo does not reproducibly prolong tumour growth delay in animals pretreated with the drug before implantation. Nevertheless, we have presented a model to investigate the role of the host in the development of drug resistance. Such a model is useful for studying the contribution of the host both in increased as well as decreased therapeutic effectiveness of other drugs. To obtain optimal therapeutic results, it will be important to minimize any contribution of the host which might lead to a decrease in the effectiveness of drugs.

C.K.L. is a MRC Postdoctoral Fellow.

We thank Dr R. Hill for constructive criticism of the manuscript, and C. Quarrington, D. Steele-Norwood, and K. Newell for technical assistance.

Supported by Grant Ca 29526 from the National Cancer Institute, NIH, USA and by a grant from the National Cancer Institute of Canada.

### References

BEGG, A.C. & SMITH, K.A. (1984). A bioassay for cyclophosphamide in blood, lung and tumour. *Br. J. Cancer*, **49**, 49.

BRAUN, D.P. & HARRIS, J.E. (1981). Modulation of the immune response by chemotherapy. *Pharmac. Ther.*, **14**, 89.

CHEEMA, A.R. & HERSH, E.M. (1971). Patient survival after chemotherapy and its relationship to *in vitro* lymphocyte blastogenesis. *Cancer*, **28**, 851.

D'INCALCI, M., BOLIS, G., FACCHINETTI, T. & 4 others (1979). Decreased half life of cyclophosphamide in patients under continual treatment. *Eur. J. Cancer*, **13**, 7.

DARNOWSKI, J.W., SAWYER, R.C., STOLIF, R.L., MARTIN, D.S. & LAU-CAM, C.A. (1985). Decreased host toxicity *in vivo* during chronic treatment with 5-fluorouracil. *Cancer Chemother. Pharmacol.*, **14**, 63.

DONELLI, M.G. & GARATTINI, S. (1971). Drug metabolism after repeated treatments with cytotoxic agents. *Eur. J. Cancer.*, **7**, 361.

GOLDIE, J.H. & COLDMAN, A.J. (1984). The genetic origin of drug resistance in neoplasms: Implications for systemic therapy. *Cancer Res.*, **44**, 3643.

GRAHAM, M.I., SHAW, I.C., SOUHAMI, R.L., SIDAU, B., HARPER, P.G. & McLEAN, A.E.M. (1983). Decreased plasma half-life of cyclophosphamide during repeated high-dose administration. *Cancer Chemother. Pharmacol.*, **10**, 192.

HILL, B.T. (1982). Biochemical and cell kinetic aspects of drug resistance. In *Drug and Hormone Resistance in Neoplasma*. Bruchovsky, N. & Goldie, J.H. (eds) p. 21. CRC Press: Boca Raton.

KALLMAN, R.F., SILINI, G. & VAN PUTTEN, L.M. (1967). Factors influencing the quantitative estimation of the *in vivo* survival of cells from solid tumours. *J. Natl Cancer Inst.*, **39**, 539.

KEMPF, R.A. & MITCHELL, M.S. (1984). Effects of chemotherapeutic agents on the immune response. I. *Cancer Invest.*, **2**, 459.

KEMPF, R.A. & MITCHELL, M.S. (1985). Effects of chemotherapeutic agents on the immune response. II. *Cancer Invest.*, **3**, 23.

LING, V. (1982). Genetic basis of drug resistance in mammalian cells. In *Drug and Hormone Resistance in Neoplasm*. Bruchovsky, N. & Goldie, J.H. (eds) p. 1. CRC Press: Boca Raton.

MANTOVANI, A., POLENTARUTTI, N., LUINI, W., PERL, G. & SPREAFICO, F. (1979). Role of host defense mechanisms in the antitumour activity of adriamycin and daunomycin in mice. *J. Natl Cancer Inst.*, **63**, 61.

MOURIDSEN, H.T., FABER, O. & SKOVSTED, L. (1976). The metabolism of cyclophosphamide: Dose dependency and the effect of long-term treatment with cyclophosphamide. *Cancer*, **37**, 665.

NOOTER, K., SONNEVELD, P., DEURLOO, J. & 4 others (1984). Repeated daunomycin administration in rats. *Cancer Chemother. Pharmacol.*, **12**, 187.

SANTOS, G.W., OWENS, A.H. & SENSEUBRENNER, L.L. (1964). Effects of selected cytotoxic agents on antibody production in man. *Ann. N.Y. Acad. Sci.*, **114**, 404.

SCHIMKE, R.T. (1984). Gene amplification, drug resistance, and cancer. *Cancer Res.*, **44**, 1735.

SCHWARTZ, H.S. & GRINDEY, G.B. (1973). Adriamycin and daunorubicin: A comparison of antitumour activities and tissue uptake in mice following immunosuppression. *Cancer Res.*, **33**, 1837.

SKIPPER, H.E., SCHABEL, F.M. Jr. & LLOYD, H.H. (1978). Experimental therapeutics and kinetics: Selection and overgrowth of specifically and permanently drug-resistant tumor cells. *Semin. Hematol*, **15**, 207.

SLADEK, N.E., DOEDEN, D., POWERS, J.F. & KRIVIT, W. (1984). Plasma concentrations of 4-hydroxycyclophosphamide and phosphoramide mustard in patients repeatedly given high doses of cyclophosphamide in preparation for bone marrow transplantation. *Cancer Treat. Rep.*, **68**, 1247.

SONNENVELD, P., STORM, A.J. & NOOTER, K. (1985). Decreased intestinal absorption of methotrexate in the rat following repeated oral dosing. *Res. Comm. Chem. Path. Pharm.*, **48**, 133.

TANNOCK, I.F. (1980). *In vivo* interaction of anti-cancer drugs with misonidazole or metronidazole: Cyclophosphamide and BCNU. *Br. J. Cancer*, **42**, 871.

VRIGNAUD, P., EGHBALI, H., HOERNI, B., ILIADIS, A. & ROBERT, J. (1985). Pharmacokinetics and metabolism of epirubicin during repetitive courses of administration in Hodgkin's patients. *Eur. J. Cancer Clin. Oncol.*, **21**, 1307.